# Arterial flow in healthy individuals and patients with hemorrhoidal disease: a Doppler ultrasound–based pathophysiological analysis

**DOI:** 10.1007/s00384-025-04951-5

**Published:** 2025-09-19

**Authors:** Gianpiero Gravante, Veronica De Simone, Roberto Sorge, Arcangelo Picciariello, Pierpaolo Sileri, Gaetano Gallo

**Affiliations:** 1 Department of General Surgery, Azienda Sanitaria Locale ASL Lecce, Casarano, Italy; 2 Colorectal Surgery Unit, IRCCS San Raffaele Scientific Institute, Faculty of Medicine and Surgery, Vita-Salute University, Via Olgettina 60, 20132 Milan, Italy; 3 Laboratory of Biometry, Department of Human Physiology, University of Tor Vergata, Rome, Italy; 4 Department of Experimental Medicine, University of Salento, Lecce, Italy; 5 Surgical Unit, IRCCS de Bellis, 70013 Castellana Grotte, Bari Italy; 6 Department of Surgery, Sapienza University of Rome, Rome, Italy

**Keywords:** Transperineal ultrasound, Doppler, Hemorrhoids, Hemorrhoidal disease, Peak systolic velocity, Goligher classification, Proctology

## Abstract

**Background:**

To evaluate arterial flow patterns in healthy individuals and patients with hemorrhoidal disease (HD) using Doppler transperineal ultrasound (TPUS), aiming to clarify the vascular contribution to HD pathophysiology.

**Methods:**

A prospective observational study was conducted on 50 healthy controls (HC) and 94 HD patients classified by Goligher grade. All underwent TPUS with Doppler assessment to record vascular patterns and quantify peak systolic velocity (PSV), end-diastolic velocity (EDV), and resistance index (RI).

**Results:**

A vascular Doppler pattern was observed in 92.6% of HD patients vs. 50% of HCs (*p* < 0.001). HD patients showed significantly higher PSV (11.1 ± 3.6 cm/s vs. 8.3 ± 2.9 cm/s, *p* < 0.001) and RI (0.8 ± 0.1 vs. 0.7 ± 0.1, *p* = 0.015), with no significant difference in EDV. Among Goligher groups, grades III and IV showed significantly elevated PSV compared to HCs. No differences were observed in EDV or RI among subgroups.

**Conclusion:**

Doppler TPUS can identify distinct hemodynamic profiles in HD patients, supporting a vascular component in HD pathogenesis. Its ability to detect subclinical alterations and distinguish severity grades may enhance diagnostic accuracy and guide tailored treatment strategies.

## Introduction

The pathogenesis of hemorrhoidal disease (HD) remains a topic of ongoing debate, with two main theories currently regarded as equally plausible. The mechanical hypothesis suggests that prolonged mechanical stress gradually leads to the prolapse of hemorrhoidal piles [1]. Contributing factors include constipation, certain postural habits (such as prolonged sitting), and reduced collagen strength commonly associated with aging. On the other hand, the vascular hypothesis focuses on altered local arteriovenous flow dynamics [2]. According to this view, engorgement of the hemorrhoidal microvasculature and progressive vessel dilation result in the formation of congested piles. This may be due to increased arterial inflow (as in hyperdynamic states), increased venous resistance (either locally, due to contraction of capillary sphincters, or systemically, such as in cirrhotic patients with portal hypertension), or a combination of both mechanisms. Both hypotheses produce local inflammation, which contributes to the worsening of symptoms over time [3].

Transperineal ultrasound (TPUS) has recently been used for the postoperative evaluation of long-term outcomes following Milligan-Morgan hemorrhoidectomy. The technique proved easy to learn, is widely available, and offers a non-invasive approach to visualizing postoperative vascular flow changes. Notably, it appears to be a highly specific tool for ruling out recurrences when the examination is negative, due to its high negative predictive value. However, this was a pilot study that did not include evaluations of vascular flow in non-operated patients or healthy individuals, was based on a small case series, and relied solely on qualitative assessments, without direct quantitative measurement of flow parameters [4].

The aim of this study was to use Doppler TPUS to assess hemorrhoidal artery flow in healthy controls (HC) and patients with varying Goligher grades of HD, in order to identify hemodynamic differences that may clarify the vascular contribution to its pathogenesis and help guide severity-based diagnostic and therapeutic strategies.

## Materials and methods

All human investigations in this study were carried out in full compliance with institutional and national ethical standards, in line with the principles outlined in the Declaration of Helsinki (1964) and its subsequent revisions. Ethical approval was obtained from the Local Ethics Committee (IRCSS Istituto Oncologico “Gabriella Serio,” approval number 2146/CEL). The study methodology and reporting adhered to the STROBE (Strengthening the Reporting of Observational Studies in Epidemiology) guidelines [5].

HC were defined as adults aged 18–80 years who presented to the general surgery clinic for non-colorectal conditions unrelated to anorectal symptoms or disease. These included benign conditions such as inguinal hernias, sebaceous cysts, or lipomas. All HC underwent a clinical evaluation to exclude any signs or symptoms suggestive of HD or other anorectal disorders and were subsequently invited to participate in the study. Patients with suspected HD who presented to the proctologic outpatient clinic during the same period were also invited to participate.

Patients were interviewed regarding the presence of common symptoms associated with HD, including bleeding, prolapse, itching, soiling/mucous discharge, and pain. In cases where prolapse was reported, additional questions were asked to allow classification according to the Goligher system [6]. Based on the responses, they were then assigned to one of the following groups: 0 (HC) or 1–4 (HD patients, classified according to the Goligher classification). If symptoms/red flags potentially indicative of colorectal cancer were present—such as rectal bleeding, tenesmus, abdominal pain, weight loss, or changes in bowel habits—a full colonoscopy or computed tomography colonography was performed (unless a recent examination was already available). Those with positive endoscopic or radiologic findings were excluded from the study and referred for further investigations/treatments. Exclusion criteria also included the presence of concomitant anorectal conditions (e.g., anal fistulas or abscesses, anal fissures, condylomas, tumors, rectocele, internal intussusception, rectal prolapse, ulcerative colitis, Crohn’s disease, or any other anoperineal disease), as well as a history of previous anorectal surgery.

The assessment of HC and HD continued with physical examination (a perineal and digital rectal examination) and a TPUS of the hemorrhoidal arteries. All TPUS examinations were carried out using the MyLab XPRO80® ultrasound platform (ESAOTE, Genoa, Italy), coupled with an mC3-11 microconvex transducer operating at 3.0–11.0 MHz. Scanning was performed via a perineal approach, placing the probe in direct contact with the anal margin using minimal pressure to optimize image acquisition without distorting local anatomy. For each subject, three sonographic views were recorded: one aligned with the sagittal plane, another with the coronal plane, and a third obtained along an oblique axis between them. Anatomical interpretation was based on criteria previously described in the literature [7, 8]. A color Doppler evaluation was also performed for each image by adjusting the volume of interest to include the internal anorectal region. Based on previously published criteria from our group, two vascularity patterns were identified and assigned to the visible vessels [4]. The “vascular” pattern was defined as the presence of a pulsatile flow in one or more clearly identifiable arteries on the scan, while the “scattered” pattern was characterized by the appearance of small, isolated color signals without a vessel-like structure or detectable pulsatility [4]. Finally, measurements of peak systolic velocity (PSV), end-diastolic velocity (EDV), and resistance index (RI) were obtained on each of the three assessed axes, and the values were recorded in a dedicated database. Clinical assessments and TPUS examinations were conducted by an experienced colorectal surgeon (GGr) with established expertise in proctologic ultrasonography.

### Outcomes

The primary outcome of this study was to describe the PSV, EDV and RI for normal subjects, asymptomatic HD, and symptomatic HD and find eventual differences between Goligher groups.

### Power analysis

The study aims to recruit at least 17 subjects in each group (HC and Goligher grades). The sample size was determined assuming a significance level (α) of 0.05 and a desired statistical power of 87. The power calculation was based on a two-sample one-sided *t*-test comparing group means (*M*1 = 6.8 vs. *M*2 = 10.7) with standard deviations (*S*1 = 1.3, *S*2 = 1.5), derived from the data published by Aigner et al. [9]. This analysis showed that 17 patients per group would provide a power of 87%. The recruitment ended when all groups reached a minimum of 17 subjects each.

### Statistical analysis

All data were inserted into an Excel database (Microsoft, Redmond, WA, USA) and analyzed with the Statistical Package for the Social Sciences Windows version 27.0 (SPSS, Chicago, IL, USA). Descriptive statistics used were the mean ± standard deviation for continuous parametric variables, the median and range for continuous non-parametric variables, and frequencies for categorical variables. Assessment of data normality was performed via histogram analysis and validated statistically using the Kolmogorov–Smirnov test. Analysis of comparison between groups was conducted with the ANOVA one-way test and Bonferroni test for multiple comparisons when variables had a continuous parametric distribution, Wilcoxon test for continuous non-parametric variables, and chi-square test for categorical variables (Fisher’s exact test if the counts in cells were inferior to 5). A *p* value less than 0.05 was considered statistically significant.

## Results

### HC vs. HD patients

**Fig. 1:**
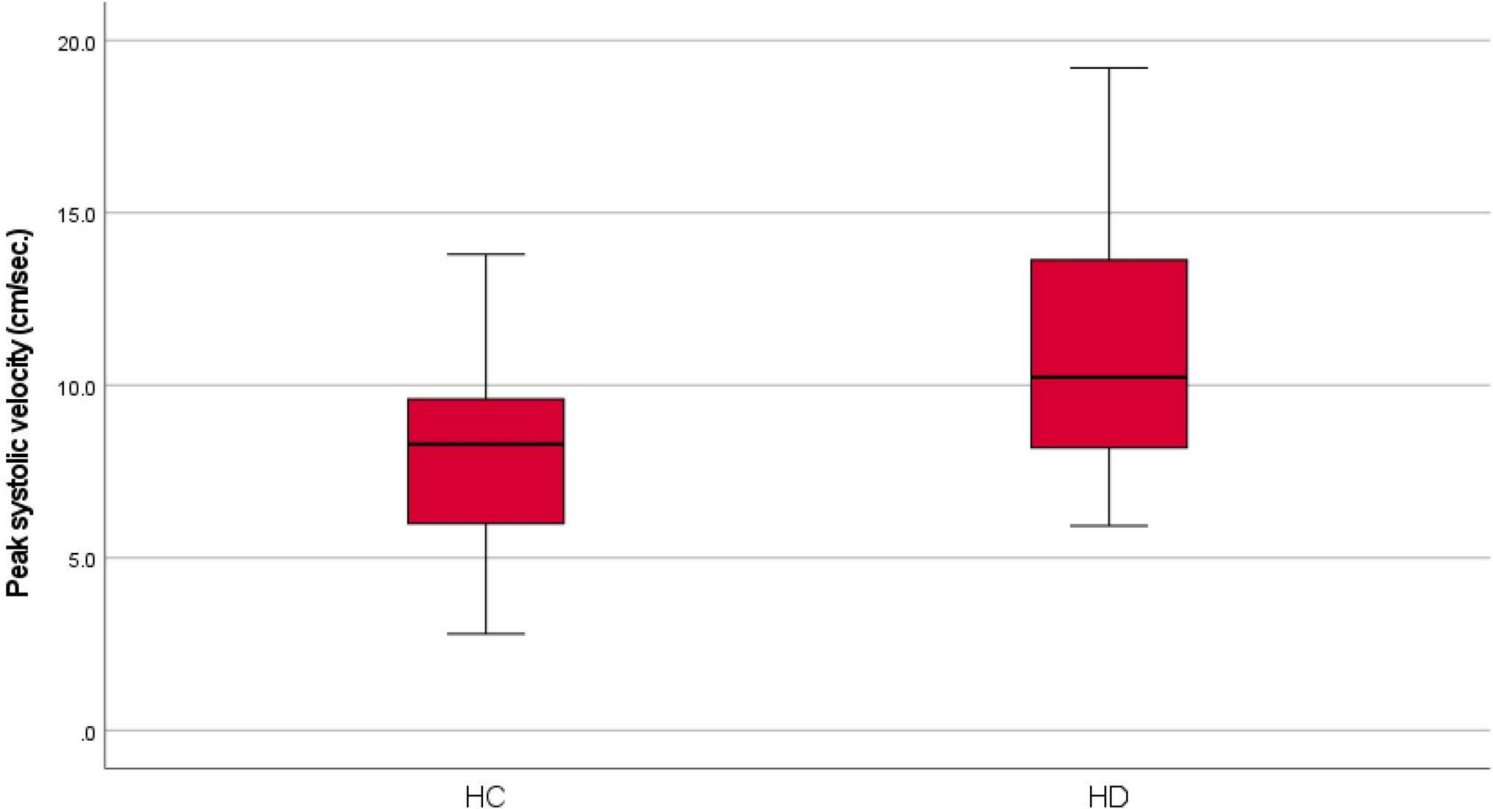
Box plots showing peak systolic velocities recorded for healthy controls (HC) and hemorrhoidal patients (HD)

Between March and May 2025, 50 HC and 94 HD patients were progressively recruited in the study. No significant differences were present for age (HC 61 ± 20 vs. HD 58 ± 15 years; Student’s *t* test *p* = 0.326) and sex (HC 39 males, 78% vs. HD 59 males, 62.8%; chi-square test *p* = 0.09). The qualitative analysis of TPUS vascularity patterns revealed a significantly higher prevalence of the vascular pattern among HD patients (*n* = 87; 92.6%) compared to HC (*n* = 25; 50%) (chi-square test, *p* < 0.001). The quantitative Doppler analysis demonstrated that PSV was significantly increased in HD patients (11.1 ± 3.6 cm/s) compared to HC (8.3 ± 2.9 cm/s; Student’s *t* test *p* < 0.001; Fig. 1). Similarly, RI was significantly increased in HD patients (0.8 ± 0.1) compared to HC (0.7 ± 0.1; Student’s *t* test *p* = 0.015). No statistically significant differences were found between the HD and HC for the EDV (2.5 ± 0.7 cm/s vs. 2.2 ± 1.4 cm/s, respectively; Student’s *t* test *p* = 0.118).

### HC vs. Goligher HD groups

**Fig. 2:**
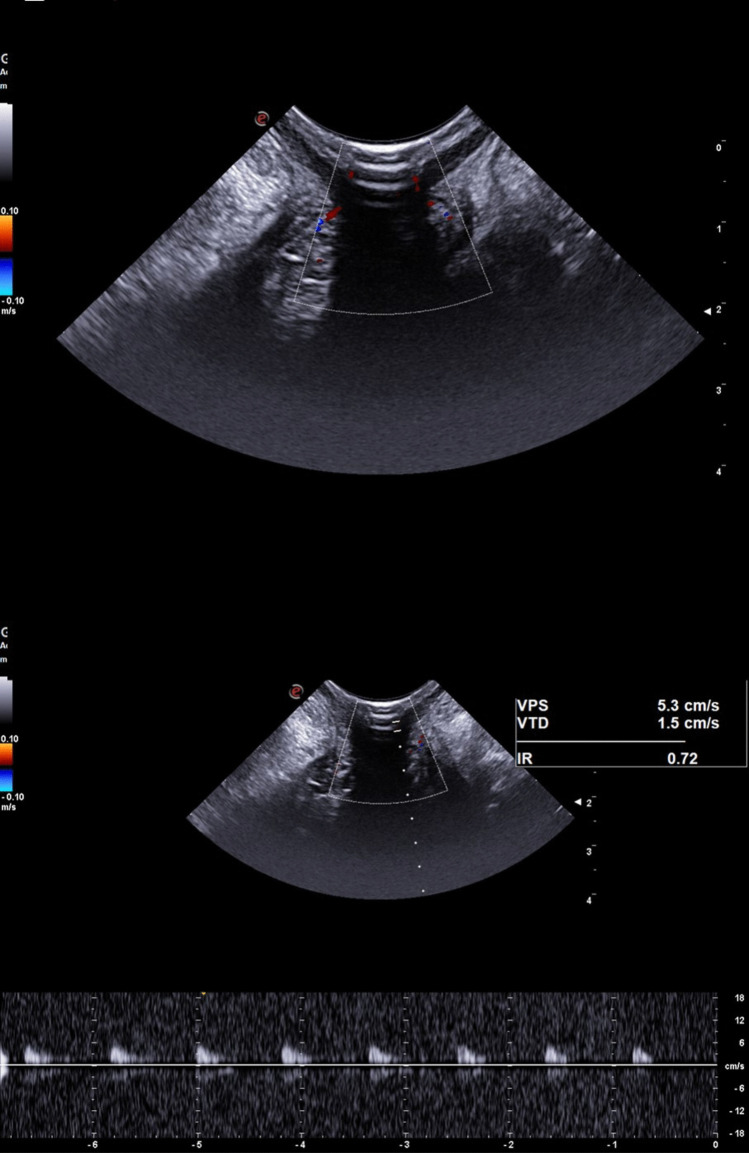
Vascular pattern (top panel) and hemodynamic parameters (bottom panel) of a healthy subject

**Fig. 3:**
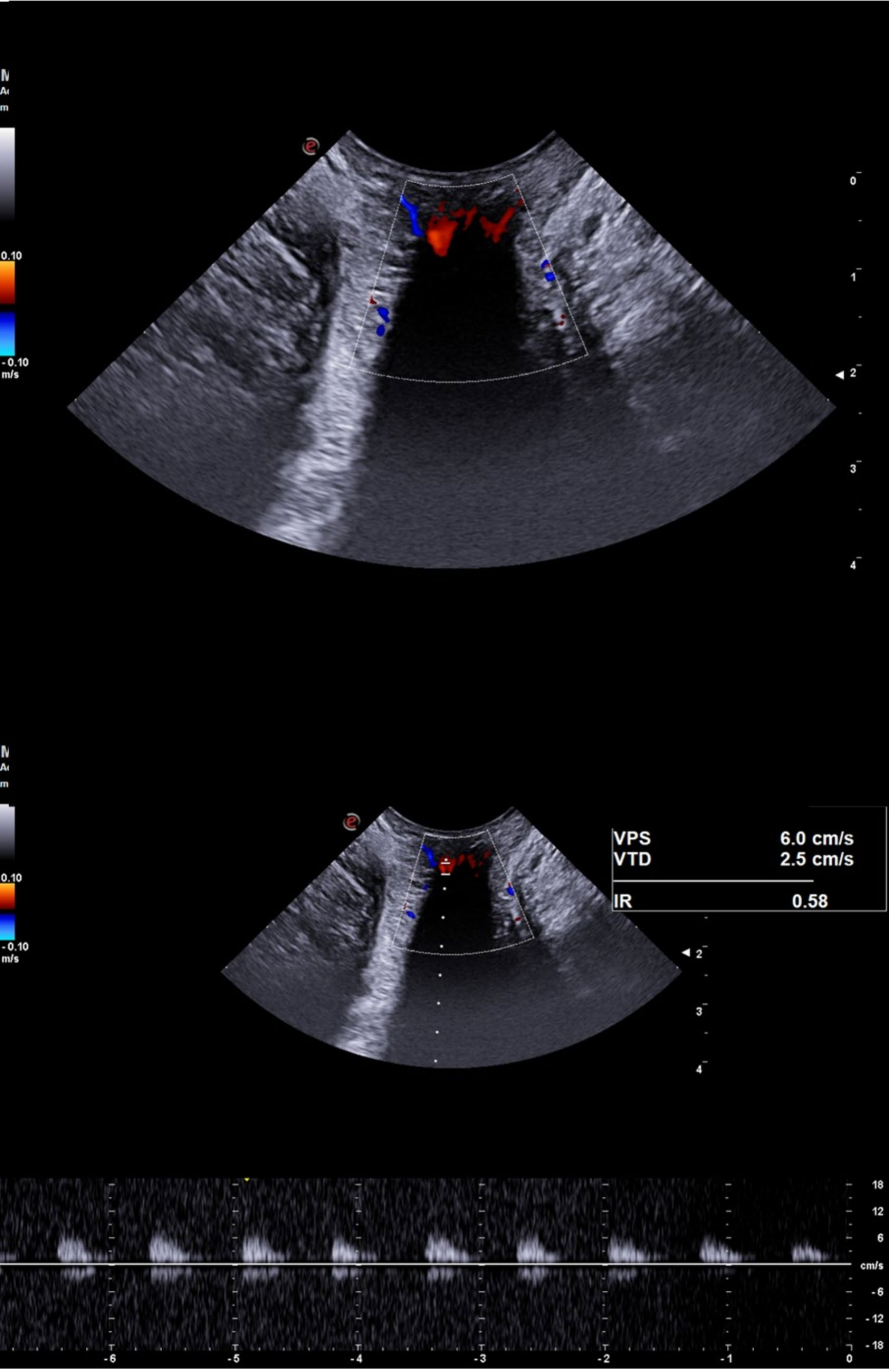
Vascular pattern (top panel) and hemodynamic parameters (bottom panel) of a Goligher 2 HD patient

**Fig. 4:**
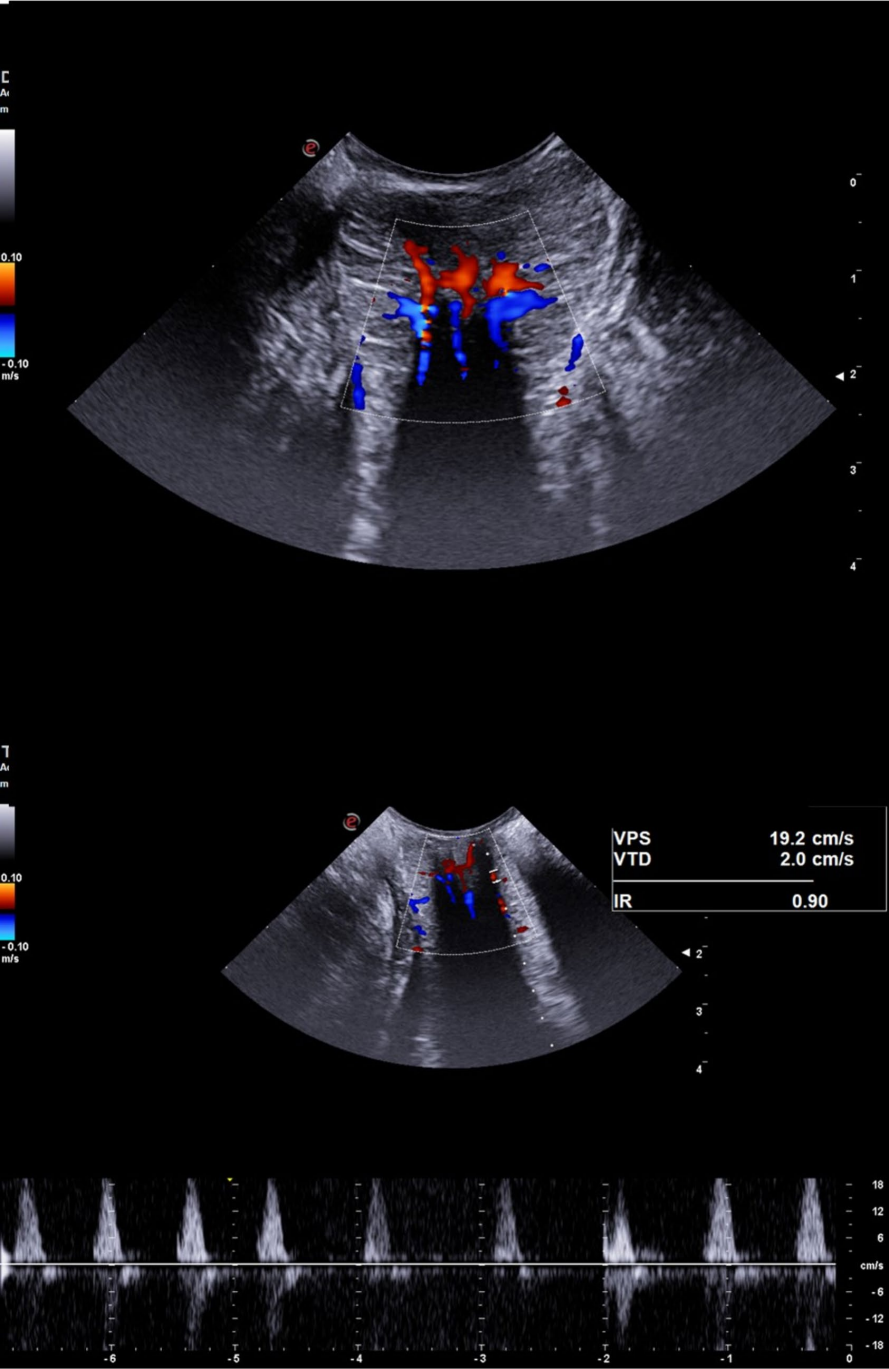
Vascular pattern (top panel) and hemodynamic parameters (bottom panel) of a Goligher 4 HD patient

**Fig. 5:**
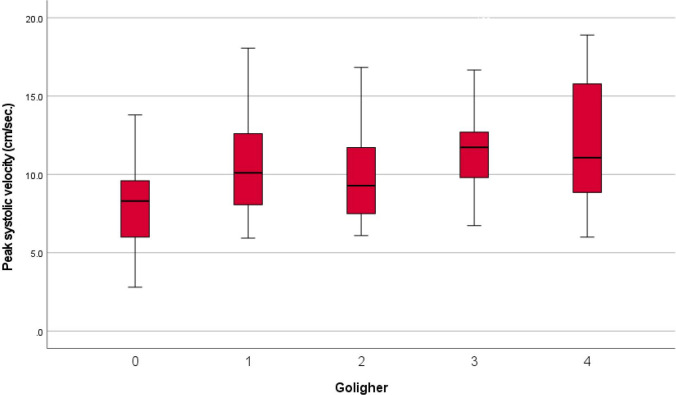
Box plots showing peak systolic velocities recorded for healthy controls and hemorrhoidal patients classified according to the Goligher degree. Goligher 0: healthy controls

The quantitative Doppler analysis demonstrated that PSV was significantly increased in patients classified as Goligher grade III and IV compared to HC (ANOVA with Bonferroni post hoc test; *p* = 0.003 and *p* < 0.001, respectively; Table 1, Figs. 2, 3, 4, and 5). In contrast, no statistically significant differences in PSV were found between HC and patients with Goligher grade I or II, nor among the Goligher subgroups themselves (i.e., between grades I vs. II and III vs. IV). Similarly, no significant differences were observed in the other hemodynamic parameters analyzed— EDV and RI—either between the Goligher groups and HC or among the Goligher subgroups.

**Table 1:** Descriptive statistics of healthy controls and patients with hemorrhoidal disease stratified by Goligher classification. Values in bold indicate statistically significant differences compared to the baseline group (healthy controls)

	Healthy controls (*n* = 50)	Goligher 1 (*n* = 22)	Goligher 2 (*n* = 28)	Goligher 3 (*n* = 17)	Goligher 4 (*n* = 27)
Age (years)	61 ± 20	58 ± 16	57 ± 16	59 ± 12	59 ± 16
Sex (males)	39 (78%)	15 (68.2%)	18 (64.3%)	11 (64.7%)	15 (55.6%)
US vascular pattern	25 (50%)	20 (90.9%)	24 (85.7%)	17 (100%)	26 (96.3%)
US scattered pattern	25 (50%)	2 (9.1%)	4 (14.3%)	0 (0%)	1 (3.7%)
Peak systolic velocity	8.3 ± 2.9	10.5 ± 3.1	10.2 ± 3.6	11.6 ± 3.3	12.3 ± 4.0
End diastolic velocity	2.2 ± 1.4	2.6 ± 0.7	2.5 ± 0.7	2.5 ± 0.8	2.4 ± 0.6
Resistance index	0.7 ± 0.1	0.7 ± 0.1	0.7 ± 0.1	0.8 ± 0.1	0.8 ± 0.1

## Discussions

The findings of our study offer several clinically relevant considerations that may help refine the current understanding and management of HD. Most notably, the Doppler analysis identified a clear hemodynamic distinction between patients with advanced HD (Goligher grades III and IV) and both HC and lower-grade HD patients (grades I and II). Specifically, PSV was significantly elevated in grades III and IV, while no significant differences were observed in grades I and II compared to HC. These results suggest that marked alterations in arterial flow are associated with more advanced stages of HD, supporting the hypothesis of a progressive vascular component in its pathogenesis. Furthermore, this threshold could prove clinically useful in stratifying patients, particularly in ambiguous cases or when symptom severity is discordant with clinical examination findings [10, 11]. The absence of significant differences in EDV and RI, both between the Goligher groups vs. HC and among Goligher groups themselves, reinforces the role of PSV as the most sensitive Doppler parameter in distinguishing HD severity.

The implications of the current findings are threefold. First, TPUS revealed a vascular pattern in 50% of HC, which rose significantly to 92.6% among HD patients. This suggests that the HC group may not be homogeneous, potentially including asymptomatic individuals with subclinical hemodynamic alterations. If confirmed by longitudinal studies, this could position TPUS as a screening tool to identify individuals at risk of HD progression—enabling earlier preventive interventions such as lifestyle modifications or medical therapy. Second, the identification of significantly elevated PSV only in grades III and IV introduces the possibility of subdividing patients into three functional categories: (1) asymptomatic HC, which may themselves be stratified based on the presence of a vascular pattern (“vascular” vs. “scattered”); (2) symptomatic patients with low-grade HD (grades I–II), who exhibit no significant hemodynamic changes; and (3) symptomatic patients with high-grade HD (grades III–IV), who demonstrate clear hemodynamic alterations. This classification could assist in resolving common diagnostic dilemmas, such as unclear prolapse dynamics or inconclusive physical findings [8], and may support the design of tailored therapeutic strategies: favoring conservative or minimally invasive options for low-grade patients and reserving more aggressive interventions for those with hemodynamic involvement [12].

Our study builds upon previous work from our group, which explored TPUS use in postoperative settings following excisional hemorrhoidectomy [4]. In that pilot study, TPUS proved to be a highly specific tool in excluding recurrences, given its high negative predictive value. However, that investigation was limited by a small sample size, lack of preoperative vascular evaluation, and a solely qualitative assessment of vascularity. The present study addresses those gaps by including a larger cohort, incorporating quantitative Doppler data, and enrolling both HC and HD unoperated patients. This diagnostic-functional integration of clinical and Doppler findings is particularly valuable in healthcare settings where access to advanced imaging technologies is limited. TPUS offers a widely available, non-invasive alternative to endoanal ultrasound, which—despite its diagnostic accuracy—is typically restricted to specialized or tertiary centers due to the need for dedicated probes and trained operators. In contrast, TPUS can be performed with conventional ultrasound equipment found in most outpatient and rural clinics, and its external approach avoids compression of soft vascular tissues, potentially providing a more physiologic depiction of blood flow. These points have also been discussed in a forthcoming correspondence article from our group, focused on imaging accessibility in proctologic practice [13].

Finally, beyond diagnosis and staging, TPUS may play a central role in guiding and assessing vascular-targeted treatments such as sclerotherapy or transanal hemorrhoidal dearterialization [14]. Its ability to detect abnormal vascular structures and quantify flow in real time may support both preoperative mapping and postoperative efficacy assessment [9]. In sclerotherapy, for example, TPUS could allow targeted injection into the most hyperemic vessels, improving outcomes and minimizing recurrence. This application is currently being explored in an ongoing prospective trial by our group.

While the results are encouraging, certain limitations of the study should be considered. First, the cross-sectional design of the study precludes any causal inference between vascular flow abnormalities and the development or progression of HD. Longitudinal studies are needed to determine whether HCs with a vascular pattern are at higher risk of developing symptomatic HD, and whether symptomatic patients without Doppler abnormalities (grade I and II Goligher) may eventually progress to the hemodynamically altered group (grade III and IV). In addition, all TPUS evaluations were conducted by the same skilled operator, ensuring procedural consistency. While this ensured technical consistency, it limits the generalizability of the findings. To address this, we are currently conducting an interobserver agreement study to evaluate the reproducibility of TPUS and to validate its applicability in broader clinical settings, including less experienced hands.

## Conclusions

This study further defines the role of TPUS as a promising non-invasive tool for evaluating HD. The identification of distinct vascular patterns and quantitative flow parameters in different patient subgroups may contribute to a better understanding of the pathophysiology of HD and assist in clinical decision-making. If confirmed in future prospective studies, TPUS could be integrated into routine proctologic assessment both for diagnostic purposes and postoperative follow-up.

## Data Availability

Data are available from the Authors upon reasonable request.
